# Milk: a postnatal imprinting system stabilizing FoxP3 expression and regulatory T cell differentiation

**DOI:** 10.1186/s13601-016-0108-9

**Published:** 2016-05-12

**Authors:** Bodo C. Melnik, Swen Malte John, Pedro Carrera-Bastos, Gerd Schmitz

**Affiliations:** Department of Dermatology, Environmental Medicine and Health Theory, University of Osnabrück, Sedanstrasse 115, 49090 Osnabrück, Germany; Center for Primary Health Care Research, Lund University, Lund, Sweden; Institute for Clinical Chemistry and Laboratory Medicine, University Hospital Regensburg, University of Regensburg, Josef-Strauss-Allee 11, 93053 Regensburg, Germany

**Keywords:** Epigenetic, Exosome, FoxO1, FoxP3, Milk, MicroRNA, mTORC1, Probiotics, TGF-β, Treg

## Abstract

**Background:**

Breastfeeding has protective effects for the development of allergies and atopy. Recent evidence underlines that consumption of unboiled farm milk in early life is a key factor preventing the development of atopic diseases. Farm milk intake has been associated with increased demethylation of *FOXP3* and increased numbers of regulatory T cells (Tregs). Thus, the questions arose which components of farm milk control the differentiation and function of Tregs, critical T cell subsets that promote tolerance induction and inhibit the development of allergy and autoimmunity.

**Findings:**

Based on translational research we identified at least six major signalling pathways that could explain milk’s biological role controlling stable FoxP3 expression and Treg differentiation: (1) via maintaining appropriate magnitudes of Akt-mTORC1 signalling, (2) via transfer of milk fat-derived long-chain ω-3 fatty acids, (3) via transfer of milk-derived exosomal microRNAs that apparently decrease *FOXP3* promoter methylation, (4) via transfer of exosomal transforming growth factor-β, which induces SMAD2/SMAD3-dependent FoxP3 expression, (5) via milk-derived *Bifidobacterium* and *Lactobacillus* species that induce interleukin-10 (IL-10)-mediated differentiation of Tregs, and (6) via milk-derived oligosaccharides that serve as selected nutrients for the growth of bifidobacteria in the intestine of the new born infant.

**Conclusion:**

Accumulating evidence underlines that milk is a complex signalling and epigenetic imprinting network that promotes stable FoxP3 expression and long-lasting Treg differentiation, crucial postnatal events preventing atopic and autoimmune diseases.

## Background

Children who grow up on traditional farms are protected from atopic diseases [1]. Early-life consumption of unboiled cow’s milk has been identified as the most protective factor for the development of atopy [2–10]. Farm milk exposure has been associated with increased numbers of CD4^+^CD25^+^FoxP3^+^ regulatory T cells (Tregs), lower atopic sensitization and asthma in 4.5-year-old children [11]. Treg cell numbers are negatively associated with asthma and perennial immunoglobulin E serum levels [11]. However, the allergy-preventive effectors of milk, which stimulate the development of Tregs remain elusive. Based on translational research we provide six potential milk-derived signalling pathways that could promote appropriate differentiation and maturation of Tregs.

## Findings

### Amino acids

Milk is the postnatal nutritional environment of all mammals that mediates immune stimulatory functions, especially long-term stable expression of FoxP3, the key transcription factor of Tregs. Milk protein provides appropriate amounts of certain insulinotropic amino acids such as essential branched-chain amino acids that induce the secretion of insulin as well as amino acids such as tryptophan that increase hepatic insulin-like growth factor-1 (IGF-1) secretion [12–21]. Both growth hormones synergistically activate the phosphoinosite-3 kinase (PI3K)-Akt pathway. Control of PI3K in Treg cells is essential for Treg lineage homeostasis and stability [22, 23]. Diminished control of PI3K activity in Treg cells reduces expression of the interleukin-2 (IL-2) receptor α subunit CD25, accumulation of FoxP3^+^CD25^−^ cells and, ultimately, loss of expression of the transcription factor FoxP3 in these cells [23]. Excessive postnatal protein intake via infant formula feeding has been demonstrated to increase infant’s serum levels of insulin and IGF-1 accelerating growth and weight gain (early protein hypothesis) [24–26]. Rapid weight gain in infancy has been linked to an increased risk of asthma [27–29].

Tregs are a developmentally and functionally distinct T cell subpopulation that is engaged in sustaining immunological self-tolerance and homeostasis. The transcription factor FoxP3 plays a key role in Treg development and function [30–33]. There is accumulating evidence that insufficient maturation and differentiation of Tregs play a key role in the development of common allergic diseases and autoimmunity [34–39].

**Fig. 1:**
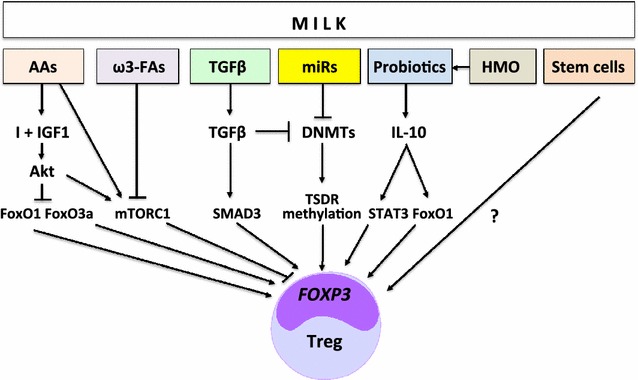
Synoptic working model for milk-induced FoxP3 expression and regulatory T cell (Treg) differentiation. The transcription factors FoxO1, FoxO3a, SMAD3 and STAT3 all enhance FoxP3 expression. Milk exosomal microRNAs and TGFβ attenuate DNA methyltransferase (DNMT) expression promoting TSDR demethylation (AAs: amino acids; ω3-FAs; ω-3 fatty acids; HMO: human milk oligosaccacharides; I: insulin; IGF1: insulin-like growth factor-1; miRs: microRNA-148a, microRNA-29, microRNA-21; mTORC1: mechanistic target of rapamycin complex 1; TGFβ: transforming growth factor-β; STAT3: signal transducer and activator of transcription 3; TSDR: Treg-specific demethylated region)

Notably, FoxP3 expression is linked to nutrient signalling via Akt-mediated phosphorylation of the transcription factors FoxO1 and FoxO3. Increased insulin/IGF-1 signalling leads to inactivation of FoxO1 and FoxO3a by their phosphorylation-dependent extrusion form the nucleus into the cytoplasm. Both FoxO1 and FoxO3a exert stimulatory effects on FoxP3 expression [40] (Fig. 1). A FoxO3a-binding motif is present in the proximal region of the *FOXP3* promoter [40]. The absence of FoxO1 severely curtails the development of FoxP3^+^ Tregs. In addition, the absence of FoxO3 exacerbates the effects of the loss of FoxO1 [41]. Thus, there is compelling evidence that increased PI3K-Akt-signalling blocks FoxP3 expression by sequestering FoxO factors [42]. FoxO transcription factors cooperatively control the differentiation of FoxP3^+^ Tregs [43]. FoxO proteins function in a Treg-intrinsic manner to regulate thymic and TGF-β-induced FoxP3 expression, in line with the ability of FoxO proteins to bind to *FOXP3* locus and control *FOXP3* promoter activity [43]. FoxO proteins are considered to play crucial roles in specifying the Treg cell lineage [43]. Genome-wide analysis of FoxO1 binding sites reveals ~300 FoxO1-bound target genes that do not seem to be directly regulated by FoxP3. These findings show that the evolutionarily ancient Akt-FoxO1 signalling module controls a genetic program indispensable for Treg cell function [44].

Upregulated PI3K-Akt signalling in the presence of sufficient amounts of branched-chain amino acids and glutamine increases the activity of the nutrient-sensitive kinase mechanistic target of rapamycin complex 1 (mTORC1) [45–47]. Milk has recently been identified as a signalling system of mammalian evolution controlling mTORC1-dependent translation [48, 49]. Enhanced mTORC1 activity was found in the brain and ileum of mice with cow’s milk allergy (CMA) [50]. Treatment with the mTORC1 inhibitor rapamycin significantly increased the mRNA expression of FoxP3 in the ileum and Peyer’s patches of CMA mice. A correlation between the extent of mTORC1-mediated S6K1 phosphorylation and FoxP3 mRNA expression in the ileum was demonstrated [50].

Taken together, the Akt-mTORC1 axis controls FoxP3 expression and differentially regulates effector and Treg cell linage commitment [43, 51–53]. It is thus conceivable that a well-balanced transfer of critical amino acids via breastfeeding controls Akt-mTORC1-mediated Treg differentiation, which may be disturbed by artificial formula feeding with high protein content [54, 55].

### Long-chain ω-3-fatty acids

Part of the asthma-protective effect is associated with the intake of raw cow’s milk and was explained by higher levels of polyunsaturated ω-3 fatty acids of farm milk [56]. Remarkably, it has been demonstrated in a mouse model of atopic dermatitis that administration of the ω-3 fatty acid docosahexaenoic acid upregulates the generation of TGF-β-dependent CD4^+^ Foxp3^+^ Tregs [57, 58]. Furthermore, fatty acids play a role in mTORC1 activation. Whereas the saturated fatty acid palmitate activates mTORC1, the ω-3 fatty acid eicosopentaenoic acid inhibits mTORC1 activation [59]. Thus, ω-3-fatty acids may not only attenuate pro-inflammatory eicosanoid biosynthesis but may exert direct effects on FoxP3 Treg activity. In fact, it has been demonstrated that Tregs transfer ω-3 long chain polyunsaturated fatty acids-induced tolerance in mice allergic to cow’s milk protein [60].

### MicroRNAs

Extracellular RNAs and especially exosomal microRNAs are regarded as most important factors involved in the regulation of the immune system [61, 62]. Human breast milk is a body fluid that is highly enriched in mRNAs and microRNAs [63]. MicroRNAs are either packaged with proteins (i.e. Ago2, HDL, and other RNA-binding proteins or wrapped in small membranous particles (i.e. exosomes, microvesicles, and apoptotic bodies) [64–67]. Human, bovine and porcine milk transfer high numbers of exosomes that contain microRNAs [68–70]. Recent evidence indicates that human milk microRNAs primarily originate from the mammary gland resulting in unique microRNA profiles of fractionated milk [71]. Recently, we hypothesized that milk transmits microRNAs (microRNA-155, microRNA-148a, microRNA-29b, microRNA-21) that may induce thymic FoxP3^+^ Treg differentiation thereby preventing the development of allergy [72]. Indeed, farm milk consumption is associated with higher *FOXP3* demethylation and higher Treg cell numbers [11]. Stable expression of FoxP3 in Tregs depends on DNA demethylation at the Treg-specific demethylated region (TSDR), a conserved CpG-rich region within the *FOXP3* locus [73–75]. In contrast, hypermethylation of the *FOXP3* gene has been associated with reduced Treg function and allergy [76, 77]. Notably, atopic individuals express lower numbers of demethylated FoxP3^+^ Tregs [78].

There are two potential mechanisms of DNA demethylation: (1) passive demethylation through inhibition of DNA methyltransferases (DNMTs) and (2) active demethylation mediated by ten-eleven-translocation (TET) 2 and 3 [79]. TET2 binding to CpG-rich regions requires the interaction of TET2 with the protein IDAX (also known as CXXC4) [80]. Intriguingly, the CXXC DNA-binding domains can bind unmethylated DNA and recruit TET2 via IDAX [81]. Thus, DNMT inhibition may favour active TET2-mediated TSDR demethylation.

Both DNMT1 and DNMT3b are associated with the *FOXP3* locus in CD4^+^ cells [82, 83]. Remarkably, DNMT1 deficiency resulted in highly efficient FoxP3 induction following TCR stimulation [82]. Importantly, DNMT1 is a direct target of microRNA-148a [84], which is abundant in bovine colostrum, mature cow’s milk, and human breast milk [68, 85, 86]. MicroRNA-148a is highly expressed in bovine milk fat and milk fat globules of human breast milk [87, 88]. MicroRNA-148a directly downregulates the expression DNMT1 and DNMT3b, whereas microRNA-21, another abundant microRNA of cow’s milk, indirectly inhibits DNMT1 expression by targeting RASGRP1 [84]. MicroRNA-29b increases dose-dependently in human serum after intake of pasteurized cow’s milk [89]. MicroRNA-29 targets DNMT3a and DNMT3b [90]. Remarkably, nucleotide sequences of microRNA-148a-3p, microRNA-29b and microRNA-21 of *Homo sapiens* and *Bos taurus* are identical (mirbase.org). Kirchner et al. [87] recently suggested that microRNAs of unprocessed cow’s milk mediate the allergy preventive farm milk effect. It is of functional importance that most milk-derived microRNAs are transported either in exosomes or milk fat globules [48, 49, 69, 70, 88–93].

It has recently been demonstrated that bovine milk microRNAs (microRNA-29b, microRNA-200c) are taken up in reasonable amounts by healthy human subjects after consumption of pasteurized cow’s milk [88]. Further evidence underlines that bovine milk exosomes are able to cross human intestinal cells and vascular endothelial cells via endocytosis [94, 95].

Notably, boiling of raw cow’s milk abolishes the allergy-preventive farm milk effect [3] and results in substantial loss of microRNA-148a-3p [87]. MicroRNA-155, another important immune regulatory microRNA of milk [72], targets suppressor of cytokine signalling 1 (SOCS1), which maintains STAT5 activity further enhancing Treg differentiation [96]. Boiling of milk may disrupt the protective lipid bilayer of milk exosomes accelerating the degradation of critical milk microRNAs. Furthermore, heat-induced alterations of exosomal membrane proteins may disturb intestinal exosome uptake. Thus, native milk-derived exosomal microRNAs via suppressing DNMTs may provide pivotal epigenetic signals stabilizing FoxP3 expression and Treg differentiation.

### Exosomal transforming growth factor-β

It has been demonstrated that exosomes of cow’s milk not only transfer microRNAs but also transforming growth factor-β (TGF-β) [97]. The TGF-β signalling pathway activates the transcription factors SMAD2 and SMAD3 [98, 99]. SMAD3 is a crucial transcription factor enhancing FoxP3 expression via binding to the conserved non-coding sequence 1 (CNS1) of *FOXP3* [100] (Fig. 1). Experimental evidence reveals that TGF-β in the context of T cell receptor (TCR) stimulation induces FoxP3 gene transcription in thymic Treg precursors, CD4^+^ CD8^−^ CD25^−^ semimature and mature single-positive thymocytes [101]. TGF-β also converts naïve T cells into inducible Treg (iTregs) and protects Tregs against apoptosis and destabilization [102]. Importantly, it has been demonstrated that TGF-β-induced expression of FoxP3 in T cells is mediated through inactivation of the kinase ERK [103]. TGF-β via inhibition of ERK activation downregulates the expression of DNMT1, DNMT3a and DNMT3b associated with increased FoxP3 expression [97]. Recently, Arntz et al. [104] confirmed that bovine milk exosomes induce FoxP3 expression and Treg differentiation in murine splenocytes. Thymus-derived exosomes as well are able to induce FoxP3^+^ Tregs in peripheral tissues [105]. Moreover, the incubation of peripheral blood mononuclear cells with isolated human breast milk exosomes increased the number of FoxP3^+^CD4^+^CD25^+^ Tregs [106]. Thus, milk-derived exosomal TGF-β acts via transcriptional control and epigenetic regulation of FoxP3 expression. It is noteworthy to mention that deficient TGF-β signalling is associated with activation of the PI3K/Akt pathway [107], which suppresses FoxO signalling.

### Bifidobaceria and lactobacilli

Human milk is a source of living bifidobacteria and lactobacilli for the infant gut [108–111]. *Bifidobacterium breve*, *B. adolescentis*, *B. bifidum*, and *Lactobacillus plantarum WLPL04* were isolated from human milk samples [108, 110]. There is accumulating evidence that probiotic bacteria generate FoxP3 T-cell responses in the small intestine [112]. *L. plantarum WCFS1*, *L. salivarius UCC118*, and *L. lactis MG1363* upregulate numbers of CD11c^+^ MHCII^+^ dendritic cells in the immune-sampling Peyer’s patches [112]. *L. plantarum*, *L. salivarius*, and *L. lactis* attenuate Th2 responses and increase Treg frequencies in healthy mice in a strain dependent manner [113]. Oral consumption of *Bifidobacterium infantis 35624* enhanced IL-10 secretion and FoxP3 expression in human peripheral blood cells pointing to the immune-stimulatory effect of bifidobacteria on FoxP3^+^ iTreg induction [114, 115]. Furthermore, it has been demonstrated that bifidobacteria stimulate TGF-β, which contributes to Treg differentiation [116].

Special attention has been paid on the critical role of the anti-inflammatory cytokine IL-10 in probiotica-induced anti-inflammatoy intestinal immune responses [110, 117, 118]. Despite heat-killing, *Lactobacillus pentosus* strain S-PT84 exhibited anti-allergic effects by modulating the Th1/Th2 balance and inducing Tregs [119]. Live and heat-killed *Lactobacillus rhamnosus* suspensions were able to induce the synthesis of different cytokines including IL-10 [120]. Heat-killed *Lactobacillus acidophilus* strain L-92 produced higher levels of Foxp3, IL-10 and TGF-β compared to control mice and suppresses allergic contact dermatitis [121]. In a bovine β-lactoglobulin-sensitized mice model, oral administration of heat-killed *L. acidophilus* exhibited increased mRNA expression of Foxp3 and TGF-β [122]. It should be noticed, that heat-treatment of probiotics may also result in a loss of immune-regulatory functions. However, the majority of studies using either native or heat-treated bifididobacteria or lactobacilli exhibits an upregulation of IL-10, TGFβ and FoxP3 [116, 119–122]. Remarkably, IL-10 potentiates differentiation of human induced Tregs via STAT3 and FoxO1 [123]. Hsu et al. [123] demonstrated that the presence of IL-10, in addition to TGF-β, leads to increased expansion of Foxp3^+^ iTregs with enhanced CTLA-4 expression and suppressive capability, comparable to that of natural Tregs. This process is dependent on IL-10R-mediated STAT3 signalling. Additionally, IL-10-induced inhibition of Akt phosphorylation and subsequent preservation of FoxO1 function are critical. In contrast to formula feeding, the presence of maternal milk in β-lactoglobulin-sensitized rat pups exhibited an immune response profile similar to that of unchallenged dam-reared rats but with greater FoxP3 mRNA expression and CD4^+^ FoxP3^+^ cells [124]. These data may explain the preventive effect of probiotics for sensitization to common food allergens associated with a reduced incidence of atopic eczema in early childhood [125]. However, due to a lack of well-designed studies convincing evidence for the prevention of allergic asthma by probiotic treatment is still missing [126, 127]. The time of onset of probiotic exposure, which is physiologically natural birth and the period of breastfeeding, may play critical roles for probiotica-induced Treg maturation.

### Milk oligosaccharides

Human milk contains large amounts of free oligosaccharides (HMOs). HMOs have been shown to exert anti-inflammatory properties, and evidence for their immune-modulatory effects is increasing [128–130]. A growing literature suggests that human milk contains viable bacteria [131]. McGuire et al. [131] postulated that human milk should be regarded as a probiotic food. Human milk oligosaccharides (HMOs) are minimally digested by the infant and are utilized by bifidobacteria [132]. One postulated function for these oligosaccharides is to enrich a specific “healthy” microbiota containing bifidobacteria, a genus commonly observed in the faeces of breastfed infants [133]. Recent studies show that some species of bifidobacteria are equipped with genetic and enzymatic sets dedicated to the utilization of HMOs promoting HMO-dependent growth of bifidobacteria [134]. Among gut microbes, the presence of enzymes required for degrading HMOs with type-1 chains is essentially limited to infant-gut-associated bifidobacteria, suggesting HMOs serve as selected nutrients for the bacteria pointing to a co-evolution between bifidobacteria and human beings mediated by HMOs [135]. Formula feeding in comparison to breastfeeding compromises the development of the physiological gut microbiome. In breastfed Rhesus infants *Bifidobacteria* and *Lactobacillus* predominated, whereas in formula-fed infants *Ruminococcus* was predominant [26]. Breastfed human infants harbour a faecal microbiota more than twice increased in *Bifidobacterium* numbers compared to formula-fed infants [136]. After formula feeding, *Atopobium* was found in significant counts and the numbers of *Bifidobacteria* dropped followed by increasing numbers of *Bacteroides* population [136]. Infant formulas containing non-digestible oligosaccharides similar to the composition in breast milk or a combination of lactic acid bacteria have been shown to harbour preventive effects towards immune-regulatory disorders [137, 138]. In fact, recent studies with whey and casein-sensitised mice showed that CD25^+^ Treg contribute to the suppression of the allergic effector response in sensitised mice induced by dietary intervention with non-digestible carbohydrates [139, 140]. Thus, it appears that milk is mammal’s early life probiotic nutrient system supporting and maintaining *Bifidobacteria* and *Lactobacillus* species that induce Tregs and control immune responses.

## Conclusions

Compelling evidence underlines that milk is not a simple food for infants but represents a sophisticated signalling network that promotes the differentiation and long-lasting maintenance of Tregs, the central players suppressing the development of allergy and autoimmunity. Milk provides an intricate metabolic, epigenetic, probiotic and stem cell-derived system that induces stable expression of FoxP3, the master transcription factor of Tregs. The critical allergy-preventive factor in cow’s milk appears to be heat-sensitive and is abolished by boiling of milk. As amino acids and the majority of long-chain ω-3 fatty acids withstand the boiling process, heat-sensitive compounds such as milk exosomes, probiotic bacteria and stem cells are the potential candidates for long-lasting Treg differentiation. In more than 200,000 million years of lactation [141, 142] the evolution of milk had plenty of time to create a sophisticated regulatory network optimizing the infant’s Treg differentiation allowing appropriate tolerance development during the postnatal period of mammalian life. In this regard, unprocessed milk should be regarded as a conditioner for tolerance development. After birth, the newborn’s immune system encounters foreign environmental antigens such as nutrients, pollens and house dust mite allergens, which via antigen-specific TCR-mediated activation induce antigen-specific Tregs. The milk-related signalling pathways presented here, may in a synergistic manner enhance antigen-specific Treg generation associated with the induction of allergen-specific immune tolerance (Fig. 1).

Most recent data underline that developing natural Treg (nTreg) cells in the thymus acquire a Treg-specific and stable hypomethylation pattern in a limited number of genes, which encode key molecules including FoxP3, essential for Treg cell function. This epigenetic change is acquired via TCR stimulation, beginning prior to FoxP3 expression. The Treg-specific DNA hypomethylated regions generally act as gene enhancers in steady state nTreg cells, contributing to the stable expression of Treg function-associated key genes including Ctla4, Il2ra, and Ikzf4 in addition to FoxP3 [143, 144]. Recent work suggests that the establishment and stability of Tregs is mediated by a number of mechanisms besides FoxP3 expression, such as epigenetic modifications, FoxO1, FoxO3a localization, expression of Eos and signalling via Neuropilin-1 [143]. To understand the pathogenesis of allergy development it is most important to characterize the immune regulatory networking of native milk. Future studies should thus focus on natural unprocessed milk and its regulatory mechanisms that support long-lasting stability of Tregs mediating livelong tolerance induction against harmless environmental antigens and autoantigens [145, 146]. We are at the beginning to understand the complex interplay of milk’s regulatory factors inducing and stabilizing the infant’s FoxP3 expression. Yet, we have no idea about the functional priority of milk-derived factors involved in Treg lineage commitment. It is thus prudent to rely on the regulatory effectiveness of our own lactation genome, which co-evolved with the lactation-associated microbiome. Therefore, we recommend breastfeeding for adequate Treg maturation and the prevention of allergic diseases of human infants. However, for mothers who are not able to provide breast milk, human donor milk might be an alternative for appropriate Treg differentiation. Countrywide access to operating human milk banks in East Germany before Germany’s unification in 1990, may explain the lower prevalence rates of atopic diseases in East versus West Germany [147, 148]. Artificial formulas still substantially differ from human breast milk. If milk exosomal microRNAs play a key role in Treg differentiation, it is of critical concern that current infant formula only contains very few human lactation-specific microRNAs [149]. The addition of bovine milk exosomal microRNAs may represent a future improvement of artificial formula feeding. Due to legal aspects and the risk of pathogen transfer, raw cow’s milk can not be recommended as an agent for allergy prevention. However, in the future, it might be possible to prepare sterilized bovine milk exosomes for this purpose. It has already been shown that bovine milk exosomes increased Foxp3 expression and attenuated arthritis in two mouse models [104].

## Abbreviations

CMA: cow’s milk allergy
CNS: conserved non-coding sequence
DNMT: DNA methyltransferase
FoxO: forkhead box class O
FoxP3: forkhead box P3
HMO: human milk oligosaccharides
IGF-1: insulin-like growth factor-1
IL-2: interleukin 2
IL-10: interleukin 10
microRNA: micro ribonucleic acid
mTORC1: mechanistic target of rapamycin complex 1
PI3K: phosphoinositide-3-kinase
SOCS: suppressor of cytokine signalling
STAT: signal transducer and activator of transcription
TCR: T cell receptor
TSDR: Treg-specific demethylation region
TET: ten-eleven-translocation
Treg: regulatory T-cell
